# Dichotomization: 2 × 2 (×2 × 2 × 2...) categories: infinite possibilities

**DOI:** 10.1186/1471-2288-10-59

**Published:** 2010-06-23

**Authors:** Karyn K Heavner, Carl V Phillips, Igor Burstyn, Warren Hare

**Affiliations:** 1 School of Public Health, University of Alberta, Edmonton, Alberta, T6G 2L9, Canada; 2 TobaccoHarmReduction.org, Saint Paul, MN, 55104, USA; 3 Department of Environmental and Occupational Health, Drexel University School of Public Health, Philadelphia, Pennsylvania, 19102, USA; 4 Department of Medicine, Faculty of Medicine and Dentistry, University of Alberta, Edmonton, Alberta, T6G 1K4, Canada; 5 Department of Math, Statistics, and Physics, University of British Columbia, Okanagan, Kelowna, British Columbia, V1V1V7, Canada

## Abstract

**Background:**

Consumers of epidemiology may prefer to have one measure of risk arising from analysis of a 2-by-2 table. However, reporting a single measure of association, such as one odds ratio (OR) and 95% confidence interval, from a continuous exposure variable that was dichotomized withholds much potentially useful information. Results of this type of analysis are often reported for one such dichotomization, as if no other cutoffs were investigated or even possible.

**Methods:**

This analysis demonstrates the effect of using different theory and data driven cutoffs on the relationship between body mass index and high cholesterol using National Health and Nutrition Examination Survey data. The recommended analytic approach, presentation of a graph of ORs for a range of cutoffs, is the focus of most of the results and discussion.

**Results:**

These cutoff variations resulted in ORs between 1.1 and 1.9. This allows investigators to select a result that either strongly supports or provides negligible support for an association; a choice that is invisible to readers. The OR curve presents readers with more information about the exposure disease relationship than a single OR and 95% confidence interval.

**Conclusion:**

As well as offering results for additional cutoffs that may be of interest to readers, the OR curve provides an indication of whether the study focuses on a reasonable representation of the data or outlier results. It offers more information about trends in the association as the cutoff changes and the implications of random fluctuations than a single OR and 95% confidence interval.

## Background

By convention, results of epidemiological analyses are often represented by a single odds ratio (OR) for a dichotomous exposure variable and sometimes with ORs for each of the confounders. An exposure variable is frequently created by dichotomizing a continuous variable such that values below a certain level, known as the cutoff, are classified as low risk and compared to those at high risk (with a value greater than or equal to the cutoff). Results are often reported for one such dichotomization, as if no other cutoffs were considered or even possible. There are several problems with representing the potentially complex relationship between an initially continuous exposure and an outcome variable by a single OR and 95% confidence interval (CI). First, this approach makes the assumption that the researcher and current and future readers are interested in the same cutoffs, which is often not the case. Second, the use of different cutoffs (as described below) across studies aimed to test the same hypothesis naturally can make it very difficult to compare results. Third, in addition to being a source of publication bias, the effect of the cutoff on the results of epidemiological studies is an overlooked area of model misspecification, even though it is known that dichotomization of a mismeasured continuous variable induces non-differential exposure misclassification [1]. Finally, when the effect estimate influences the cutoff, the snapshot of the data that is typically reported may be misleading.

The effects of publication bias are well known and have been extensively documented. However, the potential consequences of researchers' choices of which results to report from a particular study are underappreciated. The preferential reporting of particular study results, or publication bias in-situ (PBIS) [2], may skew the readers' understanding of the exposure-outcome relationship and lead to inappropriate public health policies. For example, a reader trying to summarize the literature is forced to not only deal with changing variable definitions, but cannot know whether a series of similar associations in the literature represent studies with fundamentally similar results or merely attempts to find the "same" result by analyzing the data differently. Unbeknownst to readers, a researcher having the choice of several cutoffs for creating dichotomous exposure variables, may be tempted to report only the single cutoff that best illustrates the desired outcome (e.g., the cutoff that results in an OR of 1.25 instead of 1.15) as if no other OR was obtained from the analysis, if there is no overriding reason to reject the selected dichotomization as unreasonable [3]. In contrast to typical publication bias, people interested in a particular topic have to speculate about unpublished effect estimates or other results within a given study instead of searching for whole studies that have not been published. A desired effect estimate may be obtained as a result of chance, model selection and/or varying the eligibility criteria and definitions of the outcome, exposure and covariates. In most cases, consumers of the medical and public health literature do not know if the published cutoffs were based on an a priori hypothesis about a change in the outcome at that cutoff or chosen to obtain an effect estimate that better conforms to the study hypothesis.

We focus on the consequences of selecting different cutoffs for dichotomizing a continuous exposure variable on epidemiological results and reintroduce a simple method that does not force an investigator to choose any particular dichotomization in presenting results. Specifically, we: 1) describe different strategies that are commonly used to select cutoffs for continuous exposure variables; 2) illustrate the potential effects of changing the cutoff on ORs and standard error; and 3) (re)propose a method for summarizing the results of analyses across plausible cutoffs. Our example is meant to be illustrative of a simple point, and not to investigate etiology or even a specific population. The survey sampling design variables (cluster, strata and weight) were not used, and thus the associations should not be interpreted as representing the actual U.S. population-average relationship between body mass index (BMI) and high cholesterol. Also, no attempt was made to analyze causal pathways or correct for confounders, so though we use the conventional terminology of "exposure" and "outcome," we do not mean to suggest that the association is causal.

The recommended analytical approach is not a new concept. In 1991, Wartenberg and Northridge proposed that the OR be plotted as a function of the cutoff as part of an approach to investigate the exposure distribution and its relationship to the outcome [4]. Much of their approach focused on the use of quartile-quartile (Q-Q) plots, later renamed probability-probability (P-P) plots [5,6]. They cautioned that using the OR curve to select a cutoff is a biased representation of the data and violates the assumptions needed for frequentist hypothesis testing [3,4]. Unfortunately, despite this cautionary note, the authors do provide recommendation for choosing a single cutoff using their method. ("... if one must choose a dichotomous cutpoint, or if the data seem consistent with a dichotomous exposure classification, then to be conservative in a public health sense, one should choose the largest odds ratio that is consistent with the observed data and that also provides a relatively stable estimate. Therefore, we recommend choosing the largest odds ratio value within the middle 80-90 percent of the data.") [4]. Later researchers did indeed use the P-P plots or OR curve to select a cutoff (e.g., [7,8]).

References to the 1991 article are tracked in Additional file 1. The method was useful and the P-P plots (but rarely the OR curve) were used periodically until 1994 when the method was criticized as promoting data driven cutoff selection, namely selection of the cutoff that yields the maximum OR [9]. Then the method was virtually abandoned, which is why it was not referenced in earlier versions of this work. There were only a few researchers who used Wartenberg and Northridge's method (or if others did so, they did not cite the original article). Most of the references to the 1991 article related to dichotomization in general or cutpoint bias (a type of PBIS in which researchers "Choose a few reasonable values and report results for the one that is most consistent with the investigator's a priori hypothesis.") [3]. We propose the resurrection of the OR curve (but not the more cumbersome and less flexible P-P plots), not as a method to select a cutoff but as a strategy to maximize the amount of information conveyed to readers, and thus the utility of epidemiological publications. This is particularly important at a time when synthesis of knowledge across studies is essential to formulating policy decisions. This is a timely reintroduction into the epidemiological literature as improved computational tools make the method realistic for all researchers from the novice to the veteran and readily searchable online media and post-publication peer review (e.g., including numerous blogs and epiereview.com) make it possible to monitor the use the of this method so that it is not abused again.

## Methods

### Dataset

To provide an easily-understood and replicable example, we illustrate our point using the relationship between BMI and serum cholesterol in a widely-used public database. Four waves of data (1999-2000, 2001-2002, 2003-2004, and 2005-2006) from the National Health and Nutrition Examination Survey (NHANES) were used for all examples presented here [10-13]. The sampling frame, enrolment methodology and response rates for this cross-sectional survey are described elsewhere in detail [10-13]. All analyses were conducted using SAS version 9.1 (SAS Institute, Cary, North Carolina). (The SAS code is available upon request.)

The analysis was limited to the 19,340 NHANES participants who were at least 18 years old and who had data on two continuous variables: BMI (exposure) and total serum cholesterol (outcome). The mean age of the sample was 45.9 (standard deviation = 20.1, median 44) and 48.0% were male. BMI and cholesterol were chosen because they are commonly used in epidemiological analyses, standardized (theory driven) cutoffs exist, and there is significant variation in the cutoffs used for BMI in the literature (sometimes between articles reporting analyses of the same dataset [14]http://www.tobaccoharmreduction.org/papers/heavner-phillips-heffernan-rodu-jun08.pdf). There have been several studies investigating different BMI cutoffs and there seems to be some recognition that there is not a universally appropriate cutoff [15-20].

BMI was measured in kg/m^2 ^and reported in increments of 0.01. To focus on the effect of different exposure dichotomizations, the cutoff for total serum cholesterol was held constant at ≥200 mg/dl (= case) in this analysis. This corresponds to the cutoff for "desirable" cholesterol level recommended by the CDC [21-24]. Logistic regression (PROC LOGISTIC) was used throughout the manuscript to calculate the OR and 95% CI for each cutoff.

The effect of dichotomization on the OR

#### Theory driven cutoffs

Theory driven cutoffs may be chosen based on 1) a known dose-response relationship (i.e., the threshold where biological effects are known or postulated to start); 2) a literature review of cutoffs used in previous studies; or 3) cutoffs proposed by experts, governmental agencies or non-governmental organizations. The BMI categories that are currently recommended by the CDC and World Health Organization are: underweight (<18.5), normal (18.5-24.9), overweight (25.0-29.9) and obese (>=30.0) [25,26]. These categories are a simplification, because there is evidence that the effect of BMI varies by gender, age and race/ethnicity [15,27,28]. Addressing the debate about the usefulness of these cutoffs is beyond the scope of this study, other than to note that there are many researchers who believe that other cutoffs are useful and might want to see results for them. In addition to the commonly used cutoffs of 25 and 30, those identified in a review by Kuczmarski and Flegal [15] were included as theory driven cutoffs in the present analysis.

#### Data driven dichotomizations

##### Data driven dichotomizations that are independent of the magnitude of the exposure-outcome association

In addition to the theory driven cutoffs, numerous data driven cutoffs are possible for BMI and other continuous variables. These cutoffs may be based on univariate statistics or on correlations in the data. Cutoffs may be chosen based on the *univariate distribution of the exposure variable*. The x^th ^percentile is a common cutoff [29] but any univariate statistic may be used. For this analysis, the mean, median and 75^th ^percentile were chosen, as these are commonly reported statistics in the literature. A second method is to select the cutoff based on the distribution of the exposure among the cases, typically to obtain *equal numbers of cases in the exposed and unexposed groups *to ensure equal precision in each exposure group (e.g. [30,31]). For this, the median BMI among the cases was chosen as the cutoff. Third, selecting a cutoff based on a *desired level of precision *was accomplished by calculating the OR and standard error for all cutoffs between the 25^th ^and 75^th ^percentiles of BMI in increments of 0.01. The OR and cutoff corresponding to the minimum standard error were selected. Fourth, a common method to investigate the effect of selecting various cutoffs for a predictive model is to conduct a *sensitivity analysis *and generate a receiver operating characteristic (ROC) curve (sensitivity versus 1-specificity). The area under the curve was calculated for every cutoff between the 25^th ^and 75^th ^percentiles of BMI in increments of 0.01 and graphed against the cutoff. For this method, the cutoff that maximized the area under this curve was selected as the "best" cutoff. The Youden J statistic (sensitivity + specific - 1) was also calculated for each cutoff [32].

##### Association-driven dichotomization

A particularly problematic form of data-driven dichotomization is selecting a cutoff based on *the size of the desired effect estimate*. This may mean maximizing or minimizing the OR, or selecting the OR that is closest to 1.0 if the desired result is to demonstrate that there is no association between the exposure and outcome or at what level of exposure this is true. Clearly, this can introduce large biases into research and the literature. To illustrate this case, ORs were calculated for every cutoff between the 25^th ^and 75^th ^percentiles of BMI in increments of 0.01. The cutoff with the target OR was then selected.

Recommended analytical approach - distribution of ORs for a plausible range of dichotomizations

In the absence of a strong *a priori *hypothesis, one possible analytical approach is to present cutoff/OR pairs for many cutoffs as proposed by Wartenberg and Northridge [4]. This allows both the researchers and readers to look for possible thresholds of effect and the OR corresponding to the cutoffs that they are interested in. Reporting the full range of possible cutoffs can be done with a graph of a dense set of discrete cutoffs and the corresponding ORs. This was done for the full range of cutoffs (i.e., all cutoffs from 13.37 (minimum plus 0.01) to 130.20 (maximum minus 0.01) in increments of 0.01). The ORs and 95% CIs were plotted against this full range of cutoffs. Then the graph was limited to cutoffs between the 25^th ^and 75^th ^percentiles of BMI in increments of 0.01 to illustrate the curve for a narrower range of cutoffs that includes both the overweight and obese cutoffs recommended by the CDC.

## Results

### NHANES sample

Nearly two-thirds (65.9%) of the sample had a BMI of at least 25 (overweight or obese according to the current guidelines [25,26]) and 46% had a total serum cholesterol level ≥200 mg/dl. The distributions of both BMI and cholesterol were skewed to the right. (The distributions of BMI and total serum cholesterol are illustrated in Additional file 2.)

The effect of cutoff selection on the OR

#### Theory driven cutoffs

The ORs obtained using two currently recommended cutoffs and six previously recommended sets of theory driven cutoffs are presented in Table 1. Current analyses of these data are likely to use a cutoff of 25 or 30, resulting in ORs of 1.7 and 1.2, respectively. If the contemporary recommendations for BMI cutoffs were used for analyses conducted between 1980 and the present, it would be nearly impossible to make useful comparisons, conduct meta-analyses or observe temporal trends since ORs obtained from different cutoffs would have been reported. The results might be described with similar prose, but they would, in fact, be measures of different exposures.

**Table 1:** Theory driven dichotomization

	Cutoff(s)	OR (95% CI)
**Current WHO/CDC recommendations**		
Overweight^1^	25	1.7 (1.6, 1.8)
Obese^1^	30	1.2 (1.2, 1.3)
		
**Historical cutoffs**		
1980 Dietary Guidelines^2^	Males: 26, Females: 25	1.7 (1.6, 1.8)
1984 Health United States^1^	Males: 28, Females: 35	1.2 (1.1, 1.2)
1985 NIH Consensus Development Panel, 1985 Health United States, Health People 2000^1^	Males: 27.8, Females: 27.3	1.5 (1.4, 1.5)
1985 Dietary Guidelines, 1995 Dietary Guidelines^2^	25	1.7 (1.6, 1.8)
1989 Committee on Diet and Health^2, 3^	Age specific (years): 19-24: 24, 25-34: 25, 35-44: 26, 45-54: 27, 55-65: 28, >65: 29	1.2 (1.1, 1.3)
1990 Dietary Guidelines^1, 3^	Age specific (years): 19-34: 25, >=35: 27	1.3 (1.2, 1.4)

^1 ^Comparing those with a value ≥ the cutoff to those with a value < the cutoff.

^2 ^Comparing those with a value > the cutoff to those with a value ≤ the cutoff.

^3 ^Excluded 1046 18 year olds.

ORs measured the association between BMI and high cholesterol (>=200 mg/dl) and were obtained from logistic regression.

(Historical cutoffs based on a review by Kuczmarski RJ, Flegal KM. Criteria for definition of overweight in transition: background and recommendations for the United States. Am J Clin Nutr. 2000;72(5):1074-81.)

#### Data driven dichotomizations

##### Data driven dichotomizations that are independent of the magnitude of the exposure-outcome association

A variety of data driven dichotomizations and corresponding ORs are presented in Table 2. The results obtained using the mean and median as the cutoff are similar to those obtained when the cutoff was chosen on the basis of having equal numbers of exposed and unexposed cases and minimizing the standard error. However, different conclusions about the relationship between having 'high' BMI and having high cholesterol may be reached if the 75^th ^percentile is chosen as the cutoff as this OR is approximately 1, as opposed to the median BMI (OR = 1.5).

**Table 2:** Data-driven dichotomization^1^

	Cutoff	OR (95% CI)
**Data-driven dichotomization that is not based on the exposure/outcome association**		

*Determined by the distribution of exposure variable*		
Mean BMI	28.16	1.4 (1.3, 1.5)
Median BMI	27.13	1.5 (1.4, 1.6)
75^th ^percentile for BMI	31.40	1.1 (1.1, 1.2)

		

*Determined by the distribution of outcome variable*		
Equal numbers of exposed and unexposed cases	27.84	1.4 (1.4, 1.5)

*Effect of a desired precision^2^*		
Minimizing the standard error	27.19	1.5 (1.4, 1.6)

*Maximizing the area under the curve^2^*	*25.55*^3^	*1.7 (1.6, 1.8)*

		

**Association-driven dichotomization**		
*Effect of a desired size*^*2*^		
Maximizing the OR	23.79	1.9 (1.8, 2.0)
Minimizing the OR	31.38	1.1 (1.1, 1.2)
OR closest to 1.0	31.38	1.1 (1.1, 1.2)

^1 ^Comparing those with a value ≥ the cutoff to those with a value < the cutoff. ORs measuring the association between BMI and high cholesterol (≥200 mg/dl) were obtained from logistic regression.

^2 ^Varying the BMI cutoff from the 25th (23.75) and 75th (31.40) percentiles in increments of 0.01.

^3 ^25.55 is also the cutoff with the maximum Youden's J statistic.

**Figure 1:**
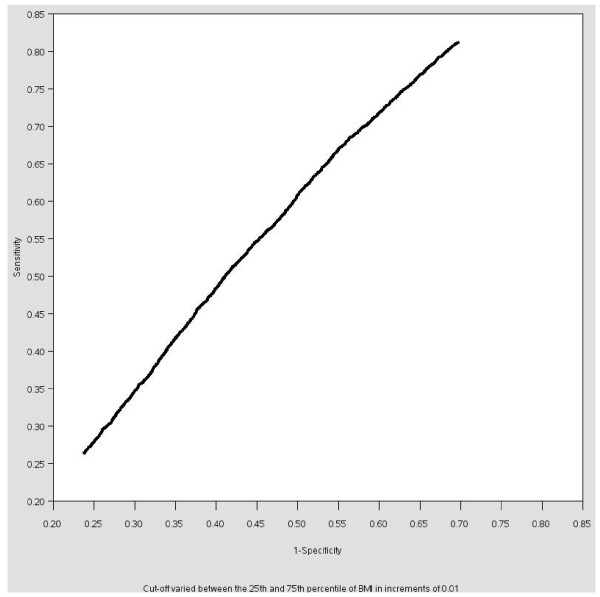
**Sensitivity analysis of the relationship between BMI and high cholesterol in the NHANES sample, 1999-2006: Receiver operating characteristic (ROC) curve**.

**Figure 2:**
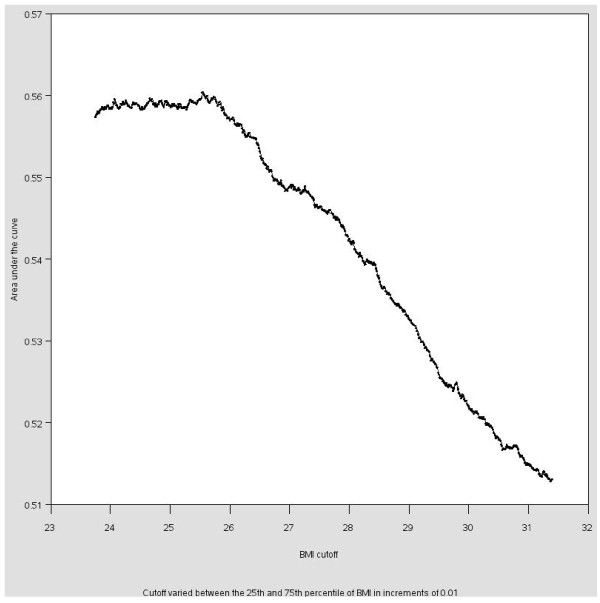
**Sensitivity analysis of the relationship between BMI and high cholesterol in the NHANES sample, 1999-2006: Area under the curve for each BMI cutoff**.

Figures 1 and 2 illustrate the ROC curve and graph of the area under the curve by cutoff for BMI cutoffs between the 25^th ^and 75^th ^percentiles. There is little difference in the area under the curve for cutoffs between 24 and 26 (with corresponding ORs varying from 1.8 to 1.6). The area under the curve decreases for cutoffs greater than 26. The area under the curve is greatest when the cutoff is 25.55, which would likely be the cutoff chosen based on this sensitivity analysis. (25.55 is also the cutoff that maximizes the Youden J statistic.) The cutoffs selected based on these four methods will likely be different for each dataset, making the results of studies that use these four dichotomization strategies difficult to compare.

##### Association-driven dichotomization

Presentation of only one OR/cutoff pair using one of the preceding methods throws away the majority of OR/cutoff pairs which are potentially informative, but does not necessarily generated a biased measure of association. But the cutoff can also be chosen based on the association. In this example, cutoffs selected between the 25^th ^and 75^th ^percentiles of BMI to maximize or minimize the OR resulted in ORs of approximately 1.9 or 1.1, respectively, which could have different implications if presented in isolation. It is apparent that the data support a wide range of ORs with approximately equal precision, enabling the investigator to select an exposure definition to obtain a simplistic interpretation of the data in such a way that it either strongly supports an association with high cholesterol or provides negligible support for the association. PBIS would result if researchers investigate such a range of cutoffs but only report one cutoff/OR pair based on their preferred association.

Recommended analytical approach

**Figure 3:**
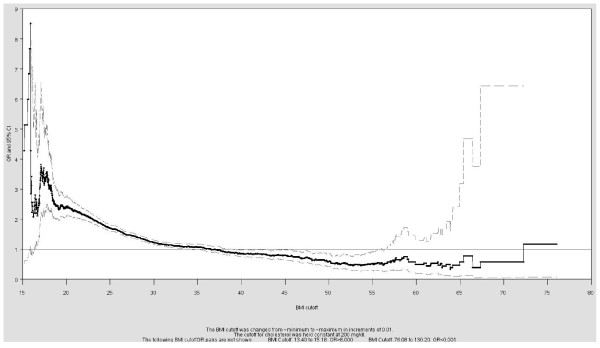
**Odds ratio curves for the relationship between high cholesterol and different BMU cutoffs in the NHANES sample, 1999-2006: The effect of changing the BMI cutoff on the OR**.

The theory driven methods and data driven methods that are not based on the desired association illustrate that there are numerous legitimate choices for dichotomization of a continuous variable. The choices motivated by the resulting association illustrate that it is easy to bias results with arguably legitimate methods using defensible cutoffs (i.e., cutoffs that would be legitimate if they were chosen for a reason other than data mining) [4]. A partial solution would be to report results for many candidate cutoffs, like in Tables 1 and 2. Wartenberg and Northridge's proposed alternative that is more complete and no more difficult to implement is illustrated in Figure 3. This graph gives the reader the OR corresponding to the cutoff that he/she is interested in and does not make the assumption that the researcher and all readers are interested in the same cutoff. It may also give readers information about possible thresholds and the stability of the OR across a range of cutoffs and may facilitate future meta-analyses by providing the ORs for a variety of cutoffs that may have been used in other studies. The approach does not make any assumptions about the shape of the association between a continuous exposure and dichotomous outcome. The potential implications of the shape of the OR curve are worthy of further investigation but are beyond the scope of the present analysis.

The value of this graph is immediately obvious even in this simple example. The "OR curve" is very unstable for cutoffs less than 20 or greater than about 55, where variation in the ORs between two consecutive cutoffs and the standard error were the greatest. In the tails, shifting a few individuals between the exposed and unexposed groups as the cutoff varies slightly results in a dramatic change in the OR. Reporting only results based on the tails, or similarly, drawing conclusions from a single study based on cutoffs in the tails would be inappropriate due to the instability in the ORs. However, those cutoffs might be of interest for some purposes, so reporting the full range avoids both over-emphasizing an unstable result and merely throwing away potentially useful information. The 95% CIs were each calculated as if they were based on an a priori hypothesis (no adjustments were made for multiple comparisons) so these should only be used by researchers investigating a specific hypothesis about the exposure at a discrete cutoff.

**Figure 4:**
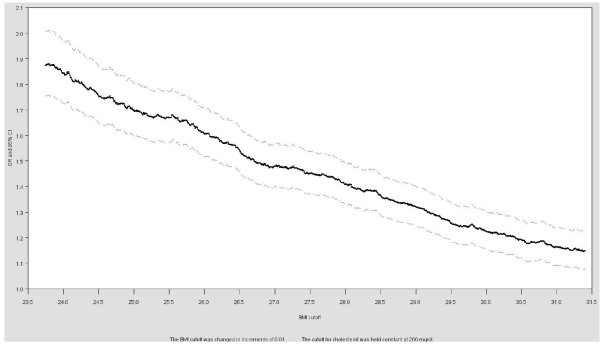
**Odds ratio curves for the relationship between high cholesterol and different BMU cutoffs in the NHANES sample, 1999-2006: The effect of changing the BMI cutoff between the 25^th ^and 75^th ^percentile of BMI on the OR**.

A representation of the OR curve that excludes the unstable tails is presented in Figure 4. This figure limits the ORs to those obtained from cutoffs between the 25^th ^and 75^th ^percentiles of BMI. The OR decreases from 1.9 to 1.1 as the cutoff increases within this range, consistent with the ROC curve. In other words, as the cutoff increases, the reference (lower BMI) group contains more people who have high cholesterol. Ideally the entire OR curve would be presented but researchers must weigh the additional information gained against the imprecise estimates and potentially more complex regression analysis (e.g., need for additional iterations or non-logistic models) in the tails of the exposure cutoff distribution.

## Discussion

If it is determined that dichotomization is the optimal or desirable variable conceptualization, there are alternatives to presenting the OR derived from a single theory or data driven cutoff. Wartenberg and Northridge's method is easily achievable using modern software, and provides a more comprehensive picture of the exposure-outcome relationship in a dataset. It offers more information about both trends in the association as the cutoff changes and the implications of random fluctuations. The present illustration demonstrates this for the relatively simple case of changing an exposure variable cutoff as well as the feasibility of presenting a more complete representation of the exposure-outcome relationship.

It has been argued that if cutoffs are not should be chosen based on an a priori hypothesis or the variable should be analyzed as a continuous variable [9]. However, in practice it is not unusual for researchers to analyze data using multiple cutoffs and report the result that conforms to their hypothesis [3]. Presenting the OR curve is a more transparent and method and does not preclude researchers from focusing the discussion a specific cutoff.

A comprehensive comparison of the advantages and disadvantages of dichotomization is beyond the scope of this paper and has been debated in detail elsewhere. Dichotomization has been proposed as a method to guard against misspecification of the disease model, but even in this application it does not appear to be a universally advantageous solution [33,34]. One of the benefits of dichotomization is that the methods and results are often more understandable and inherently useful to the average researcher and consumer of epidemiology, including clinicians, than is the reporting of continuous functions (including splines (e.g. [35]), polynomial regression, and other methods used to fit a curve to an outcome as a function of a continuous independent variable). Dichotomization can usefully summarize the effect of an exposure, particularly when supplemented with other analytic strategies [34]. We propose the presentation of OR curves as one such analytic strategy.

This method is fairly simple and flexible. It can be used for either categorical or continuous outcomes, unlike the P-P plots. Similar, albeit more complex, methods may also be used if it is important or desirable to use a polychotomous exposure variable or convert continuous covariates or outcomes to categorical variables. It would be more difficult to comprehensively report the interaction of changing cutoffs for several variables, though a third axis or multiple OR curves may be presented. It is important to realize that while this strategy solves some problems, it does not address other problems from categorizing continuous variables, such as non-differential misclassification in the imputed categorical variables due to measurement error in the continuous variable [1]. This is an analysis of the available data, not the subjects' true BMI and cholesterol values so measurement error was not taken into account.

A common exposure variable, like BMI, which generates high interest and numerous plausible cutoffs, emphasizes how much useful information is lost when a single cutoff is presented. For less-studied variables and idiosyncratic studies the greater importance of reporting all possible cutoffs might be able to reduce bias. Running multiple models and presenting only one exposure cutoff and the corresponding OR, as if no other models were considered, may introduce bias to reported study results [3]. The desire to obtain a parsimonious result that reduces the representation of complex social and biological relationships to a single number may have resulted in the preferential publication of ORs on one side of the OR curve.

The dataset used in this work is an unusually large sample for an epidemiological study, so the variation is almost entirely due to true differences in the association when different cutoffs are used, rather than random error. Cutoffs between the 25^th ^and 75^th ^percentile were focused on to illustrate the variation in ORs that is possible from a relatively narrow range of cutoffs in the middle of the range of exposure values. In the middle of the OR curve, there were only slight changes in the OR between cutoffs. The OR curve for a small sample is likely more similar to the tails of the presented OR curve, where moving a few subjects from one category to another strongly affects the association and the same OR may be obtained from disparate cutoffs. In that case, the potential for bias is greater as the associations are less stable and random variation may produce an outlier OR for a narrow range of cutoffs.

## Conclusion

The influence of cutoff selection is invisible to the reader and may be a mystery to the researcher if cutoff selection is not discussed and only the results obtained from a single cutoff are presented. Presenting only one preferred cutoff/OR pair when multiple such pairs were investigated results in a large amount of potentially useful knowledge being discarded and exchanges unbiased random error for non-random error. Frequentist test statistics are typically reported, but they are no longer meaningful since the error is no longer random [36]. This is rarely acknowledged by researchers and few readers are aware of the problem or the resulting pattern of bias. The post-publication solution would be to make data available for reanalysis so that other researchers can run models using their own choices of cutoffs and other modeling decisions. Until such transparency of data and results is the standard of practice, reporting the full range of possible ORs offers a partial solution and may help reverse the prevalent myth and belief that there is a "right answer," or "correct OR" that can be derived from a single study.

## Abbreviations

CI: confidence interval; NHANES: National Health and Nutrition Examination Survey; OR: odds ratio, PBIS: publication bias in-situ.

## Competing interests

At the time that this research was conducted, Drs. Heavner and Phillips were funded by an unrestricted grant to the University of Alberta, School of Public Health, from U.S. Smokeless Tobacco Company. Dr. Burstyn is funded by a Population Health Investigator salary award from the Alberta Heritage Foundation for Medical Research. This research was investigator initiated and the funders played no role in it. Dr. Phillips has built much of his career on questions of how epidemiologic research results are misleading as currently reported, and in particular has openly argued that the biggest such problem is publication bias in situ (PBIS), though there is limited agreement on this point. Thus, he has the incentive to produce analyses that demonstrate the potential implications of PBIS.

## Authors' contributions

The study was initiated by CVP and all the authors contributed to generating the methods. KH conducted the data analysis. All authors contributed to writing the manuscript and have read and approved the final manuscript.

## Pre-publication history

The pre-publication history for this paper can be accessed here:

http://www.biomedcentral.com/1471-2288/10/59/prepub

## Supplementary Material

Additional file 1 **Appendix 1**. Studies that referenced the 1991 Wartenberg and Northridge study. Click here for file

Additional file 2 **Appendix 2**. BMI and total serum cholesterol in the NHANES sample, 1999-2006 (n = 19,340). Click here for file
