# Supplementary figures and images for: Dichotomization: 2 × 2 (×2 × 2 × 2...) categories: infinite possibilities
Source: BMC Med Res Methodol. 2010 Jun 23;10:59. doi: 10.1186/1471-2288-10-59 (PMC2902492; doi:10.1186/1471-2288-10-59)

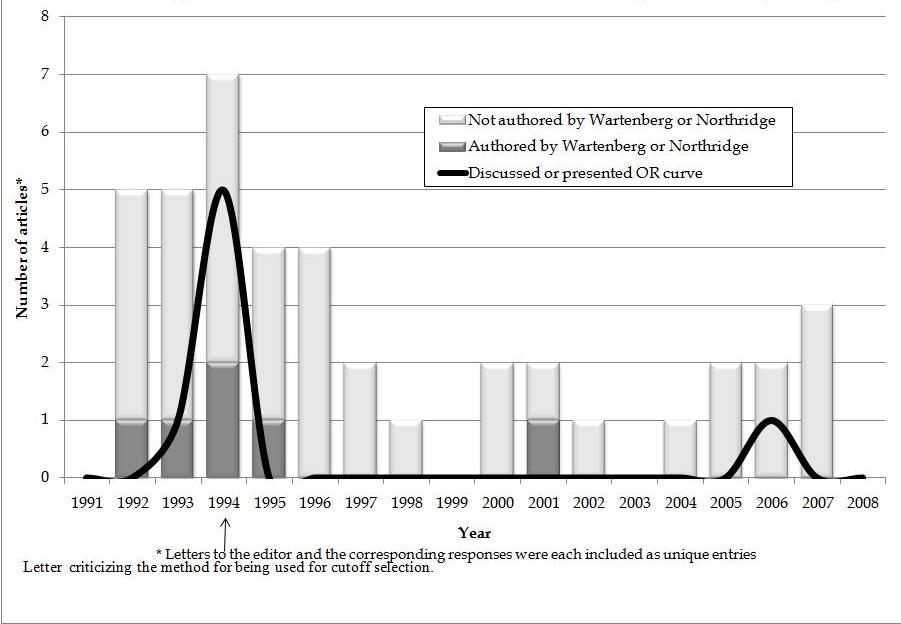

Supplement: Additional file 1 — Appendix 1. Studies that referenced the 1991 Wartenberg and Northridge study. [file 1471-2288-10-59-S1.JPEG]

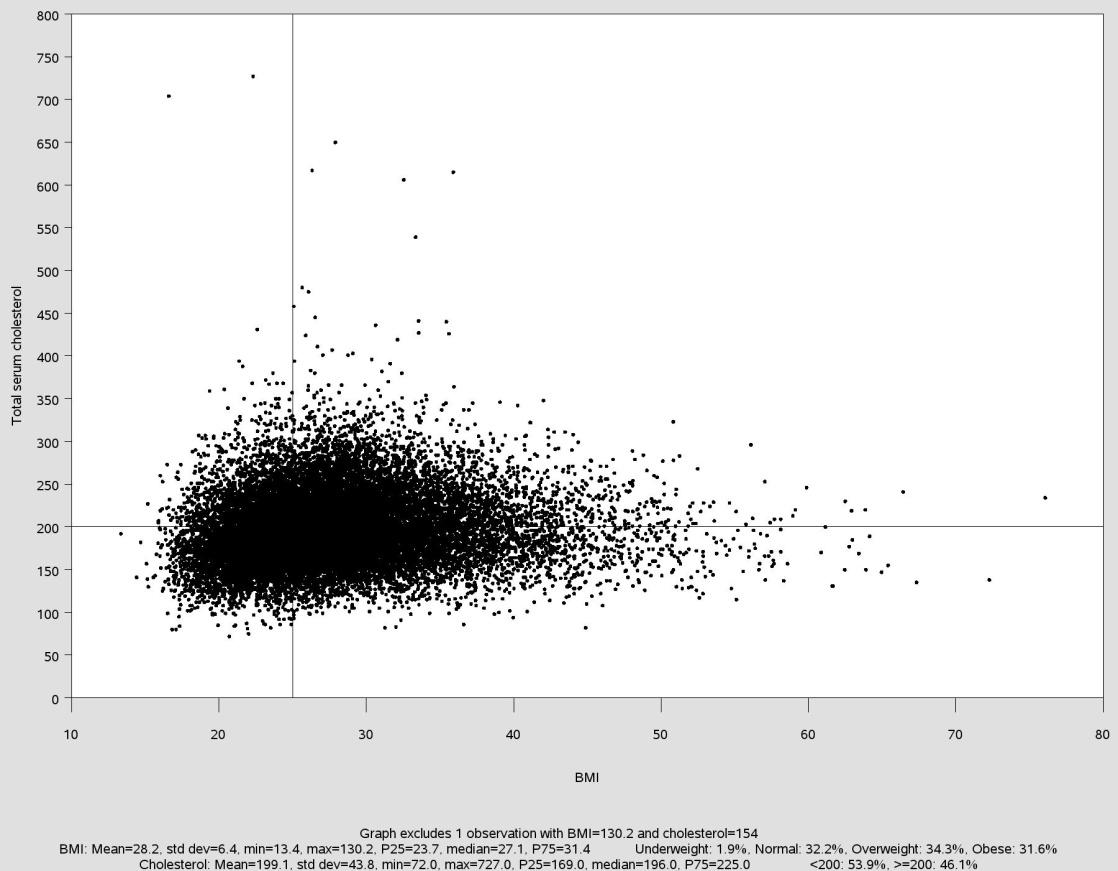

Supplement: Additional file 2 — Appendix 2. BMI and total serum cholesterol in the NHANES sample, 1999-2006 (n = 19,340). [file 1471-2288-10-59-S2.JPEG]
